# Exploring the Change in Redox Reactivity of UO_2_ Induced by Exposure to Oxidants in HCO_3_^–^ Solution

**DOI:** 10.1021/acs.inorgchem.3c00682

**Published:** 2023-05-02

**Authors:** Junyi Li, Xianjie Liu, Mats Jonsson

**Affiliations:** † Department of Chemistry, School of Engineering Sciences in Chemistry, Biotechnology and Health, KTH Royal institute of Technology, SE-10044 Stockholm, Sweden; ‡ Laboratory of Organic Electronics, Department of Science and Technology, Linköping University, Norrköping, SE-60174, Sweden

## Abstract

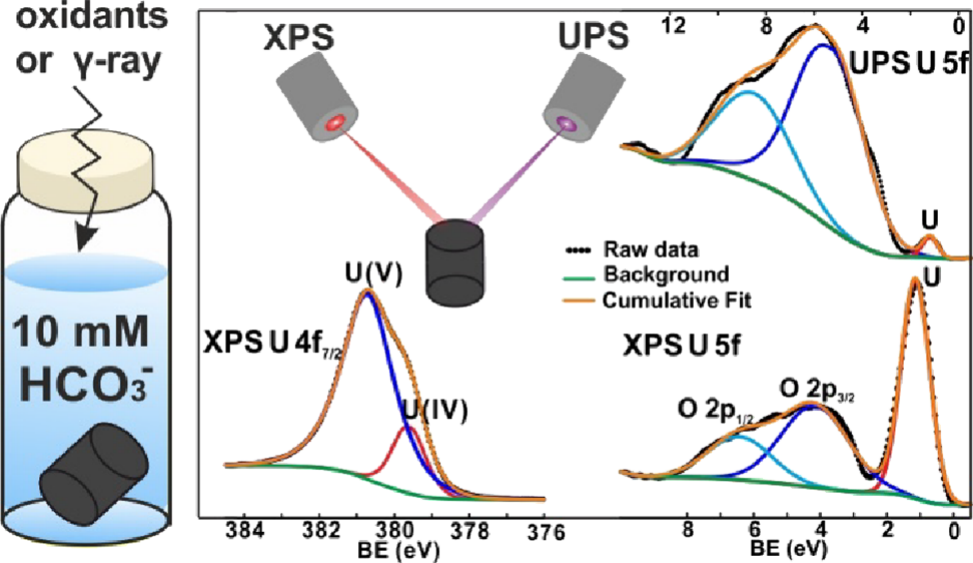

Understanding the possible change in UO_2_ surface reactivity after exposure to oxidants is of key importance when assessing the impact of spent nuclear fuel dissolution on the safety of a repository for spent nuclear fuel. In this work, we have experimentally studied the change in UO_2_ reactivity after consecutive exposures to O_2_ or γ-radiation in aqueous solutions containing 10 mM HCO_3_^–^. The experiments show that the reactivity of UO_2_ toward O_2_ decreases significantly with time in a single exposure. In consecutive exposures, the reactivity also decreases from exposure to exposure. In γ-radiation exposures, the system reaches a steady state and the rate of uranium dissolution becomes governed by the radiolytic production of oxidants. Changes in surface reactivity can therefore not be observed in the irradiated system. The potential surface modification responsible for the change in UO_2_ reactivity was studied by XPS and UPS after consecutive exposures to either O_2_, H_2_O_2_, or γ-radiation in 10 mM HCO_3_^–^ solution. The results show that the surfaces were significantly oxidized to a stoichiometric ratio of O/U of UO_2.3_ under all the three exposure conditions. XPS results also show that the surfaces were dominated by U(V) with no observed U(VI). The experiments also show that U(V) is slowly removed from the surface when exposed to anoxic aqueous solutions containing 10 mM HCO_3_^–^. The UPS results show that the outer ultrathin layer of the surfaces most probably contains a significant amount of U(VI). U(VI) may form upon exposure to air during the rinsing process with water prior to XPS and UPS measurements.

## Abstract 2

XPS results show that U(V) is the main oxidation state of uranium after UO_2_ was exposed to either oxidants or γ-radiation in HCO_3_^−^ solution. The stoichiometric ratio of U/O of oxidized UO_2_ was calculated as UO_2.3_ under all the oxidizing conditions applied. UPS results indicate that the outer ultrathin layer of oxidized UO_2_ contains a significant fraction of U(VI).

## Introduction

Nuclear power is a significant contributor to the total energy supply in many countries. It is regarded as a clean energy source in the sense of CO_2_ emissions and therefore has an important impact on sustainable development.^1,2^ However, the obvious drawback is the inevitable production of highly radiotoxic spent nuclear fuel. For UO_2_-based fuel (the most common type in commercial reactors), the spent nuclear fuel contains approximately 95% UO_2_ and 5% radioactive fission products or heavier actinides.^3,4^ Since the start of the nuclear power era, more than 400,000 t of spent fuel has been generated. About two-thirds is kept in storage while the other third has been reprocessed.^5^ Currently, the spent nuclear fuel is temporarily stored in storage pools or in dry casks. Permanent storage of spent nuclear fuel is an essential component of the nuclear waste management system in several countries. Many countries plan to place the spent nuclear fuel in geological repositories where the hazardous material will be protected by engineered and natural barriers for periods exceeding 100,000 years.^6−10^ Given the extremely long time periods during which the repository must remain safe, extensive safety assessments are required before taking a repository into use. Groundwater intrusion is a potential scenario that must be considered. When groundwater comes into contact with the spent nuclear fuel, the radiolysis of water produces both oxidants (OH^·^, H_2_O_2_, HO_2_^·^, and O_2_) and reductants (e_aq_^–^, H^·^, and H_2_).^11,12^ In general, UO_2_ has very low solubility in water. However, after the U(IV) is oxidized by the radiolytic oxidants to U(VI), the solubility of the fuel matrix will significantly increase. The solubility is further enhanced by the formation of highly soluble complexes between U(VI) and Lewis base ligands (e.g., CO_3_^2–^, OH^–^, O_2_^2–^).^13−16^ The concentrations of HCO_3_^–^ in the groundwater with a depth relevant to repositories (ca. 500 m) are in the range 0.1–10 mM.^17−21^ The radiation-induced oxidative dissolution of the fuel matrix (UO_2_) will result in radionuclide release, and therefore, understanding UO_2_ matrix dissolution under repository conditions is of major importance for the safety assessment of a deep geological repository.^22^

In general, at HCO_3_^–^ concentrations as high as 10 mM, the UO_2_ surface is assumed to be kept free from oxidized UO_2_ and the stoichiometry is expected to be UO_2.0_. However, in some fairly recent studies of H_2_O_2_-induced oxidative dissolution of UO_2_ pellets in 10 mM HCO_3_^–^, it was discovered that the redox reactivity of UO_2_ decreases with increased H_2_O_2_ exposure.^23^ H_2_O_2_ has been shown to react with UO_2_ via catalytic decomposition to produce O_2_ and H_2_O and by oxidizing the surface. The observed change in reactivity only applied to the latter reaction pathway. Raman spectroscopy shows that the surface is significantly oxidized after the exposure of H_2_O_2_ even in solutions with 10 mM HCO_3_^–^.^23^ This implies that there might be a stable (or semi-stable) oxidized phase formed on the UO_2_ surface that can alter the redox reactivity of the material. Similar studies for other radiolytic oxidants have not been presented as far as we know.

Torrero et al.^24^ studied the dissolution of UO_2_ in carbonate-free solution under different O_2_ partial pressures. It was shown that there is no significant difference in the steady-state release rate of uranium between exposure to 5% O_2_/N_2_ and 100% O_2_ at pH 8.6 (a pH close to what is expected in 10 mM HCO_3_^–^). XPS analysis performed on the UO_2_ after exposure revealed a relatively high percentage of U(VI) with a stoichiometry close to UO_2.6_ in the solid surface layer. Furthermore, de Pablo et al. studied O_2_-induced dissolution of UO_2_ in 0.1–50 mM CO_3_^2–^/HCO_3_^–^ medium and concluded that only a contribution from U(IV) can be observed based on the XPS results.^25^

In this work, we have explored how consecutive O_2_ and γ-radiation exposures in aqueous solutions containing 10 mM HCO_3_^–^ affect the reactivity of UO_2_. The potential surface modification connected to the exposures was analyzed using X-ray photoelectron spectroscopy (XPS) and ultraviolet photoelectron spectroscopy (UPS). For the surface analysis, specimens exposed to either O_2_, H_2_O_2_, or γ-radiation in aqueous solutions containing 10 mM HCO_3_^–^ were used. The XPS and UPS results were analyzed using the following methods: (1) deconvolution of U 4f_7/2_ peak; (2) energy difference between the U 4f_5/2_ peak and its corresponding satellite peaks; (3) peak center and FWHM of the O 1s peak; (4) peak area ratio between O 2p_3/2_ and U 5f.^26−30^ The observed impact of exposure on the reactivity of UO_2_ is discussed in view of the XPS and UPS results.

## Experimental Section

**Caution**! Although the radioactivity of natural uranium (prior to its use in a nuclear reactor) is low, safety precautions regarding work with radioactive materials should be followed. Experiments involving uranium should only be conducted by trained staff and take place in facilities appropriate for the handling.

All solutions were prepared using Milli-Q water (18.2 MΩ cm), and all chemicals used were of reagent grade unless otherwise stated. Hydrogen peroxide 30% (Merck) and sodium bicarbonate (NaHCO_3_, Merck) were used to prepare stock solutions. The UO_2_ pellets (geometrical surface area of approximately 352 mm^2^) were supplied by Westinghouse AB.^22^

The concentrations of U(VI) in solution were measured spectrophotometrically using the Arsenazo III method,^31^ where uranyl reacts with the Arsenazo III reagent forming a complex in acid media. The absorbance of the complex is measured at λ = 653 nm using a Thermo Scientific Genesys 20 spectrophotometer. During the measurement, 1.5 mL of diluted sample was mixed with 60 μL of 1 M HCl and 40 μL of 16 wt % Arsenazo-III reagent in a cuvette. The detection limit of U(VI) is 0.22 μΜ.

### UO_2_ Pellet Surface Pre-Washing

Before the dissolution experiments, the UO_2_ pellets were washed in de-aerated 10 mM NaHCO_3_ to remove the pre-oxidized phase. The washing steps were carried out according to following procedures: Each UO_2_ pellet was first rinsed with 10 mM NaHCO_3_, and then the pellet was placed in a glass vessel with several glass pearls on the bottom of the vessel. Then, 35 mL of 10 mM NaHCO_3_ was added into the glass vessel and sealed by a rubber septum with N_2_ purging for 20 min. The bicarbonate solution was then replaced, and the purging continued for 24 h. After that, the solution was replaced again and the purging continued for 20 min in fresh bicarbonate solution. After washing, the solution was replaced by 40 mL of 10 mM NaHCO_3_ to be used in the dissolution experiments.

### Consecutive O_2_ Exposure Experiments

One washed UO_2_ pellet with 40 mL of 10 mM NaHCO_3_ was placed in a glass vessel sealed by a rubber septum. Several glass pearls were placed on the bottom of the glass vessel to increase the surface area of the UO_2_ pellet exposed to the solution. The O_2_ gas was continuously purged into the solution through a thin glass tube. Three consecutive O_2_ exposure experiments were performed at room temperature, and each exposure lasted for 360 h or more. The concentration of U(VI) was monitored as a function of time. For each U(VI) measurement, a 0.8 mL aliquot was taken from the solution. Since O_2_ purging can accelerate the evaporation of water, the volume of the solution was recorded after each sampling for further volume compensation calculations. Between individual exposures, the exposed pellet was washed by 10 mM NaHCO_3_ according to the washing procedure described above.

### Consecutive Irradiation Exposure Experiment

For each experiment, one washed UO_2_ pellet with 40 mL of 10 mM NaHCO_3_ and several glass pearls were sealed by a rubber septum in a glass vessel. γ-radiation is emitted from a Cs-137 gamma source (Gammacell 1000 Elite, MDS Nordion) with a dose rate of 0.11 Gy s^–1^ determined by Fricke dosimetry.^11^ The sample was exposed to γ-radiation at room temperature for three consecutive times. The concentration of U(VI) was monitored as a function of time. For each U(VI) measurement, 2 mL aliquots were taken from the solution. Before the exposure and after each sampling, the solution was purged with N_2_ for 15 min to remove gaseous radiolysis products and then sealed tightly with septum and parafilm. Between individual exposures, the exposed pellet was washed with 10 mM NaHCO_3_ according to the washing procedure described above. Table 1 summarizes the exposure conditions of the UO_2_ pellets used in the dissolution experiments.

**Table 1:** Summary of the Exposure Conditions of UO_2_ Pellets Used in the Experiments

UO_2_ pellet	exposure conditions
UP-1	4 O_2_ exposures
UP-2	3 γ-irradiation exposures
UP-3	2 O_2_ exposures +2 γ-irradiation exposures
UP-4	3 O_2_ exposures +2 γ-irradiation exposures
UP-5	3 O_2_ exposures +2 γ-irradiation exposures

### XPS and UPS

The XPS and UPS experiments were carried out in an ultrahigh vacuum surface analysis system (base pressure of 5 × 10^–10^ mbar) with a SCIENTA ESCA200 hemispherical electron analyzer. The electrons were excited by a monochromatized Al Kα source (1486.6 eV) for XPS measurements and a standard He discharge lamp He II (40.8 eV) source for UPS measurements. The spectrometer was calibrated with the reference of the Fermi edge (0.0 eV) and Au 4f_7/2_ peak position (84.0 eV). The total energy resolution of XPS was set so that the FWHM (full width at half-maximum) of the clean Au 4f_7/2_ line (at the binding energy of 84.00 eV) is 0.65 eV. The total energy resolution of UPS was about 0.1 eV, as estimated from the width of the Fermi level. All spectra were recorded at normal emission and room temperature.

Note that the samples used in the XPS measurements were not the same samples used in the dissolution experiments. The samples used in the XPS measurements were UO_2_ slices cut from one UO_2_ pellet. This was done to minimize the initial difference between the samples and have samples that fit the instrument. This original UO_2_ pellet was sintered in the same batch as the pellets used in the dissolution experiments. The cut UO_2_ slices were exposed to different oxidizing conditions by repeating the procedures described for the dissolution experiments presented above. The exposure details are listed in Table 2. Prior to XPS/UPS analysis, the samples were rinsed with pure water and dried in a glovebox. During the transport from glove box to spectrometers, the samples were sealed in microcentrifuge tubes (Polypropylene) filled with Ar from the glovebox.

**Table 2:** Samples Used in XPS and UPS and Exposure Conditions Prior to the Measurements

UO_2_ slices	exposure conditions before XPS characterizations
US – O_2_ ref	stored in a glovebox for a total of 45 days
US – O_2_ exp	3 O_2_ exposures for a total of 45 days
US – H_2_O_2_ ref	Stored in glove box for a total of 30 days
US – H_2_O_2_ exp	3 H_2_O_2_ exposures for a total of 30 days irradiation
US – irradiation ref	stored in a glovebox for a total of 10 days
US – irradiation exp	3 γ-irradiation exposures for a total of 10 days

### XPS and UPS Analysis

Data mining for the XPS and UPS raw data was performed by Thermo Avantage Software (ver. 5.9931). The background was subtracted based on the “smart” function in the software. This “smart” background subtraction is based on the Shirley background subtraction with the additional constraint that the background intensity should never exceed the raw data intensity at any range. The raw data was smoothed by Savitzky–Golay filtering with a window size of 1 eV and polynomial of 4. Peak deconvolution was based on the Gaussian–Lorentzian product function. Specific principles of U 4f, O 1s, O 2p, and U 5f peak deconvolution are described in their corresponding results part.

## Results

### Uranium Dissolution

**Figure 1:**
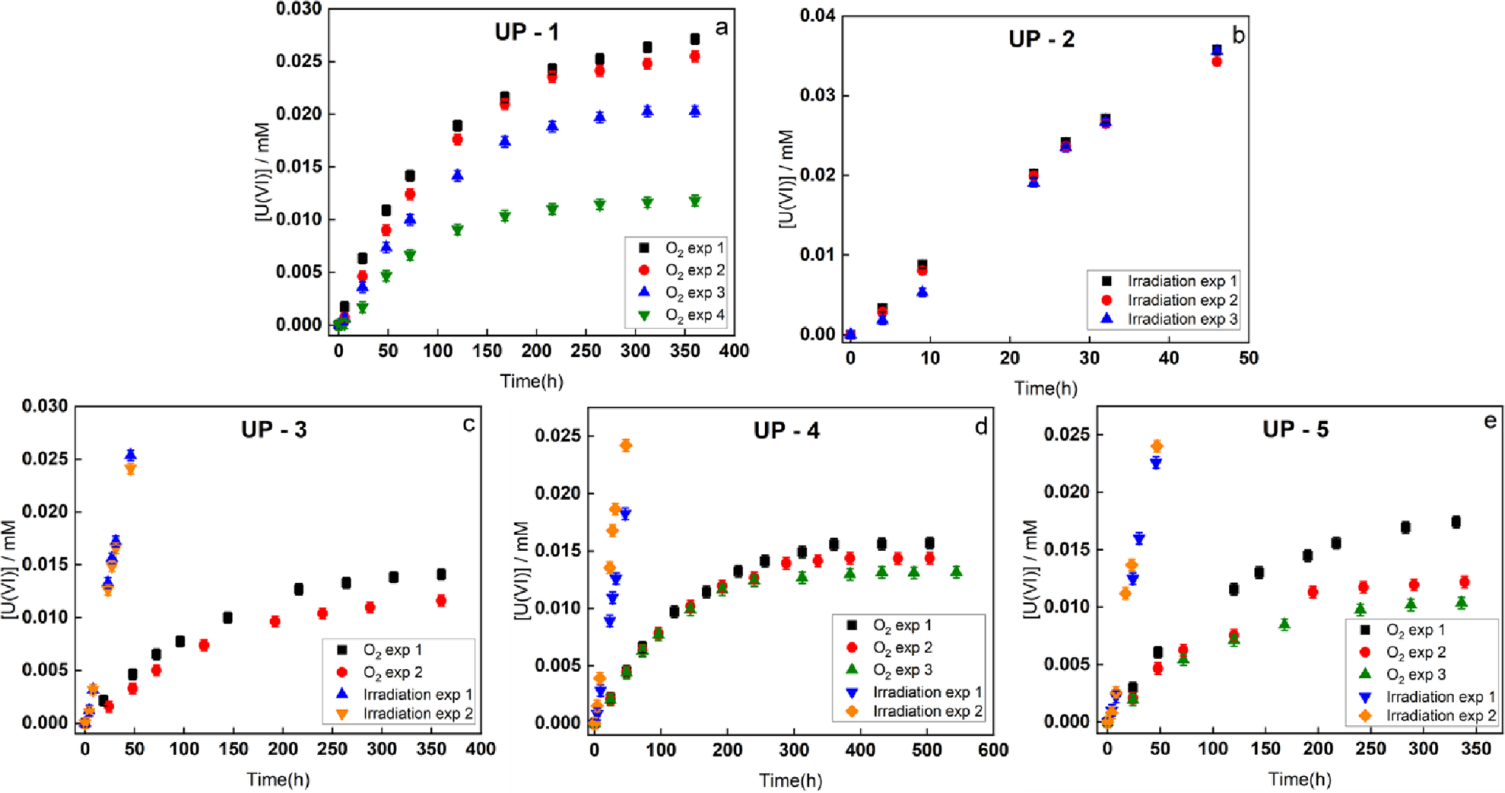
Concentration of U(VI) as a function of time in solutions containing UO_2_ pellets in 10 mM NaHCO_3_ exposed to O_2_ or γ-radiation.

Figure 1a–e shows the dissolution of uranium from five individual UO_2_ pellets consecutively exposed to oxidizing conditions in 10 mM NaHCO_3_. Each measurement of the U(VI) concentration was performed in a doublet. The average number of the two measured U(VI) concentrations was used in Figure 1. The standard deviation of the two measurements is less than 0.5 μM, and error bars are included in Figure 1. The oxidizing conditions were achieved through exposure to O_2_ and γ-radiation in various combinations. Since the solution volume in the reaction vessel will gradually decrease due to sampling and evaporation of water, volume compensated concentrations (normalization) are used throughout the work. Details of the normalization are shown in the Supporting Information.

Figure 1a shows the dissolution behavior of the UO_2_ pellet (UP-1) in 10 mM NaHCO_3_ under 4 consecutive O_2_ exposures. The O_2_ exposure is achieved by continuously purging the solution with O_2_. In this type of exposure, the concentration of O_2_ in solution is constant (approximately 1.22 mM determined by Henry’s law at 1 atm pressure^32^). Hence, if the reactivity of O_2_ toward UO_2_ remains constant, we would expect a constant rate of dissolution as long as there are no solubility limitations. As can be seen, within every single exposure, the uranyl concentration increases with exposure time. However, the dissolution slows down with exposure time. It is important to note that the concentrations are still very low compared to the solubility limit of U(VI) under the present conditions. When comparing the consecutive exposures, it is evident that the rate of oxidative dissolution also decreases for each exposure although the initial rate of oxidative dissolution of subsequent exposures is significantly higher than the final rate in the previous exposure. The rationale for this is probably that the surface is altered in a way that reduces the redox reactivity, but that this alteration is at least partly reversible through the washing step between exposures. The same trend was observed also for UP-3–5 during the O_2_ exposures. The change in reactivity of the UO_2_ pellets can be due to an oxidative alteration of the surface. Figure 1b shows the dissolution of uranium from a UO_2_ pellet in 10 mM NaHCO_3_ under 3 consecutive exposures to γ-radiation. In this system, a number of different one- and two-electron oxidants are formed. The main oxidants are OH^•^, CO_3_^•–^ (produced upon reaction between OH^•^ and HCO_3_^–^), and H_2_O_2_. These oxidants can oxidize U(IV) to U(V) or U(VI). As can be seen, the uranium concentration increases linearly with irradiation time and the trend is more or less identical for consecutive exposures. The radiation exposures presented for UP-3–5 reveal the same trend. Given the results for O_2_ exposure presented above and the previously published results on H_2_O_2_ exposure,^23^ the results for consecutive radiation exposures are somewhat unexpected. However, it should be kept in mind that the exposures to O_2_ and γ-radiation are quite different. In the O_2_ exposure experiments, the O_2_ concentration of approximately 1.22 mM (determined by Henry’s law at 1 atm pressure) is maintained constant through the continuous purging with O_2_. Any change in rate of oxidative dissolution must then be attributed to a change in the reactivity of the UO_2_ surface. During exposure to γ-radiation in a γ-source with a constant dose rate, the rate of oxidant production is constant. In such a system, a steady state will be reached where the rate of oxidant production is equal to the rate of oxidant consumption. If the surface reactivity changes in this system, the steady-state concentration of the oxidants will change until the rate of oxidant consumption is again equal to the rate of radiolytic oxidant production. Hence, we cannot expect to observe a difference in dissolution behavior even if there is a change in surface reactivity.

### XPS and UPS

The potential change in oxidation state of UO_2_ pellet slice surfaces after three consecutive exposures to either O_2_, H_2_O_2_, or γ-radiation was studied by XPS. Reference samples for each exposure condition were stored in 10 mM NaHCO_3_ in a glovebox (O_2_ ≤ 0.1 ppm) for the same duration of time as the respective exposures.

**Figure 2:**
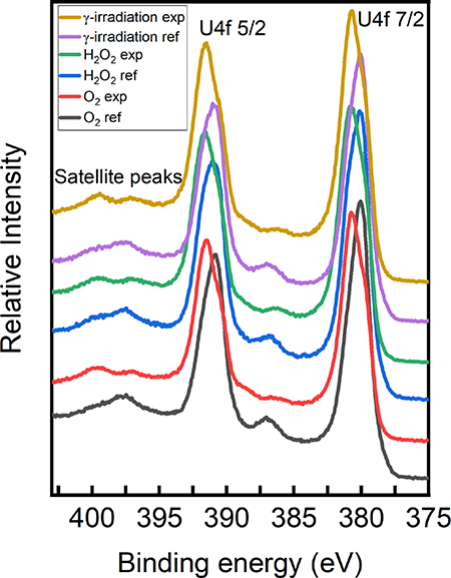
U 4f XPS spectra of the UO_2_ slices exposed to various oxidizing conditions and reference samples.

Figure 2 shows the narrow scans of U 4f. As can be seen, the measured U 4f_7/2_ and U 4f_5/2_ in the reference samples are close to 380.0 and 391.0 eV, respectively, which is in line with previous results.^26,27,29^ Both peaks shift to higher binding energy after exposure to O_2_, H_2_O_2_, or γ-radiation, indicating that the surface was oxidized. There are four methods that can indicate oxidation states of uranium from XPS spectra: (1) deconvolution of the U 4f_7/2_ peak; (2) distance between the U 4f_5/2_ peak and its corresponding satellite peaks; (3) peak center and FWHM of the O 1s peak; and (4) peak area ratio between O 2p_3/2_ and U 5f.^26−30^ The present work will utilize all four methods to investigate the oxidation state of uranium. Note that only method 1 was used for quantitative analysis.

### Deconvolution Principle

The spectral features of the U 4f_5/2_ and U 4f_7/2_ core level lines are very sensitive to probe the chemical state of uranium. Since the shape of U 4f_5/2_ peaks is affected by the satellite peaks generated from U 4f_7/2_ peaks, deconvolution was only performed for U 4f_7/2_ peaks. Each U 4f_7/2_ peak was assumed to contain three components including U(IV), U(V), and U(VI).^29,30,33^ The peak positions in the U 4f_7/2_ peak deconvolution process were chosen on the basis of the peak positions of pure U(IV), U(V), and U(VI) materials. The deconvolution principle for mixed valence oxidized UO_2_ samples is according to the guidance in ref (34), in which a fixed Gaussian–Lorentzian characteristic, one variable but identical FWHM for each component peak, and a floating peak center is suggested. In this work, a 20% Gaussian–Lorentzian characteristic was used for all U 4f peaks deconvolution, which is similar to the ratio used in ref (35) (15%).^35^ FWHM values of U(IV), U(V), and U(VI) peaks in oxidized UO_2_ samples were all fixed to 1.40 eV, which is the same as the value used in ref (36). Generally, the peak centers of component peaks are directly related to the chemical environment in XPS spectra. Since the chemical environments (the number of oxygen bond to uranium, and the crystal structure) of U(IV), U(V), and U(VI) can change upon oxidation, the peak centers of U(IV), U(V), and U(VI) were allowed to float close to reference reported values, i.e., ∼380 U(IV), ∼381 U(V), and ∼382 eV U(VI).^33−36^ Also, the peak center distance between U(IV) and U(V) was fixed to 1.00 ± 0.02 eV. No U(VI) peak close to 382 eV can be identified by the software (Avantage, ver. 5.9931) in all the U 4f spectra. Examples of forcibly adding the U(VI) peak in the U 4f peak deconvolution was shown and discussed in detail in the Supporting Information (Figure S7). The absence of U(VI) can be attributed to the fairly high HCO_3_^–^ concentrations used in the exposures and the fact that the samples were carefully rinsed with pure water prior to XPS analysis. The cumulative fits are shown in individual deconvolution spectra.

**Figure 3:**
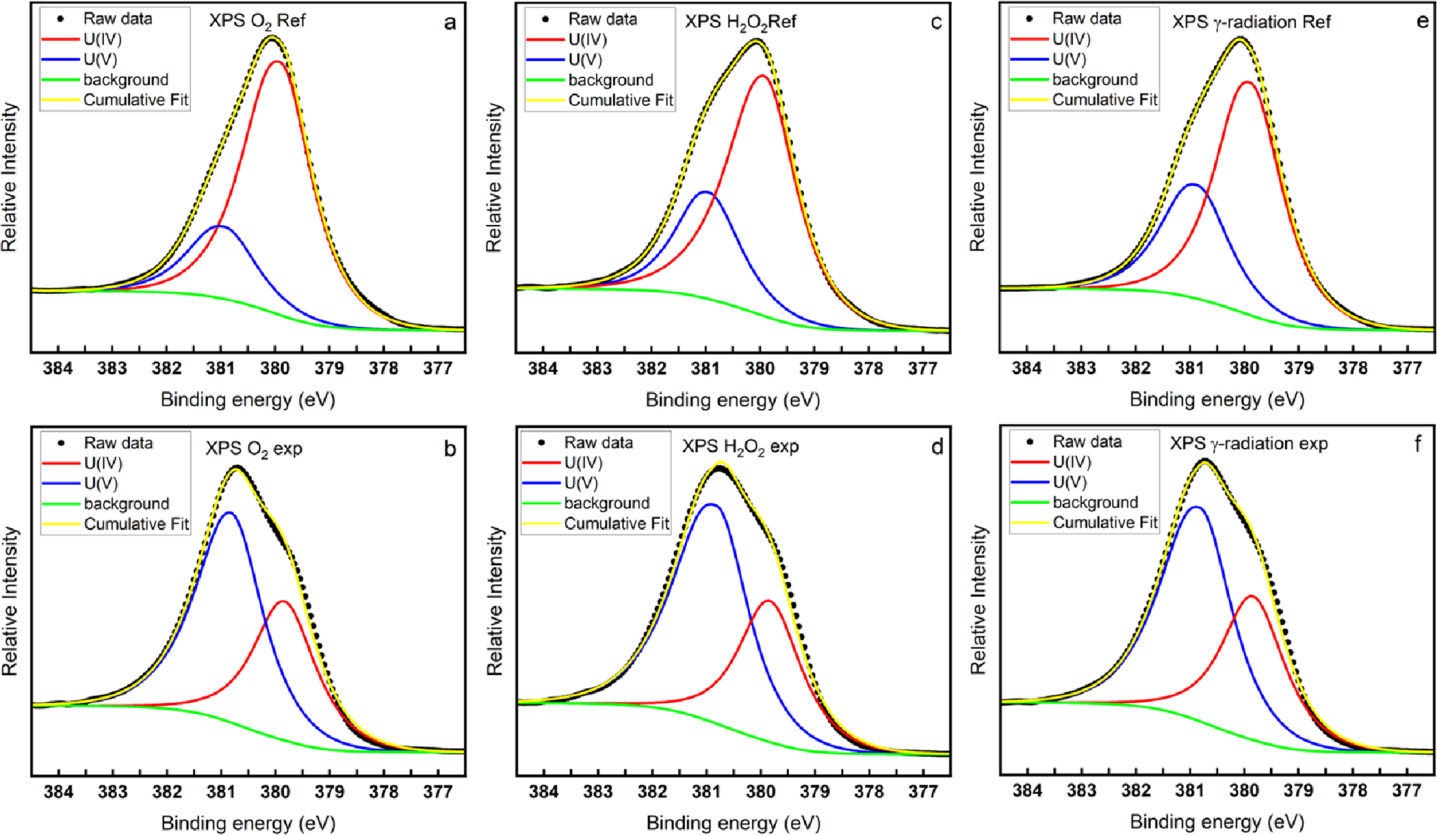
Deconvolution of U 4f_7/2_ into U(IV) 4f_7/2_ and U(V) 4f_7/2_.

Figure 3 shows the deconvolution of U 4f_7/2_ peaks of samples exposed to O_2_, H_2_O_2_, or γ-radiation compared to their corresponding reference samples. The deconvoluted U(IV) peak is marked in red and the U(V) peak is marked in blue. It is clear that the peak area of U(V) increases after exposure. By calculating the peak area ratio between U(V) and U(IV), the stoichiometric ratio of O/U can be obtained. Interestingly, the calculated stoichiometric ratio of O/U of the samples after exposures to all the three oxidizing conditions is close to UO_2.32_. The uranium peak assignment in the U 4f_7/2_ spectra in this work is in line with refs (37−39) that reported that the uranium on a UO_2.33_ surface is a combination of U(IV) and U(V) without U(VI). The peak positions, FWHM of the component U(IV) and U(V) peaks, and the calculated peak area ratio between U(V) and U(IV) are summarized in Table 3. The percents of different states of uranium are shown in Table S1.

**Table 3:** Summary of the Deconvolution of U 4f_7/2_ and the Calculated Stoichiometric Ratio of O/U

exposure condition	U(IV) peak position	U(V) peak position	U(IV) FWHM	U(V) FWHM	area ratio between U(IV) and U(V)	calculated stoichiometry
O_2_ ref	379.91	380.93	1.40	1.40	3.55	UO_2.11_
O_2_ exp	379.84	380.82	1.40	1.40	0.62	UO_2.31_
H_2_O_2_ ref	379.92	380.94	1.40	1.40	2.44	UO_2.15_
H_2_O_2_ exp	379.84	380.82	1.40	1.40	0.55	UO_2.32_
irradiation ref	379.89	380.87	1.40	1.40	2.03	UO_2.17_
irradiation exp	379.84	380.82	1.40	1.40	0.6	UO_2.31_

The U 4f_5/2_ peak positions and the positions of its satellite peaks as well as the distance between the U 4f_5/2_ peak and its corresponding satellite peaks are summarized in Table 4. The distance between the U 4f_5/2_ peak and its corresponding satellite peaks indicates the oxidation states of uranium. In the O_2_ exposure reference sample, the distance is 6.65 eV, which is close to the reported value of U(IV) (6.9 ± 0.2).^29^ Note that there is another inconspicuous satellite peak in the H_2_O_2_ and radiation exposure reference samples with the distance toward the U 4f_5/2_ peak close to 8.45 eV, indicating that U(V) is present. Moreover, the reported U 4f_5/2_-satellite distance value for pure U_2_O_5_ is 7.9 eV.^26^ There are two satellite peaks observed in the O_2_, H_2_O_2_, and radiation exposed samples, with the U 4f_5/2_–satellite distance of approximately 5.6 ± 0.15 and 7.95 ± 0.2 eV. The peak with lower binding energy is the U(IV) satellite peak and the other peak with higher binding energy is the U(V) satellite peak. Noteworthily, the satellite peak with higher binding energy is more pronounced than the other satellite peak, indicating the dominance of U(V). The close peak position of the more intense satellite peaks between the oxidized samples indicates the close stoichiometric ratio of O/U. The U 4f_5/2_–satellite distances for U(VI) are at 4.4 and 9.9 eV, respectively.^26^ As can be seen, these satellite peaks are not present in any of the samples indicating the absence of U(VI) in all the measured samples.

**Table 4:** Summary of the U 4f_5/2_ Peak Position and the Distance between U 4f_5/2_ and the Satellite Peaks

exposure condition	U4 f_5/2_ peak position (eV)	satellite peak (S1) position (eV)	satellite peak (S2) position (eV)	distance between U 4f_5/2_ and S1 (eV)	distance between U 4f_5/2_ and S2 (eV)
O_2_ ref	390.80	397.45		6.65	
O_2_ exp	391.45	397.00	399.60	5.55	8.15
H_2_O_2_ ref	391.10	397.50	399.85	6.40	8.75
H_2_O_2_ exp	391.55	397.20	399.45	5.65	7.90
irradiation ref	391.05	397.50	399.50	6.45	8.45
irradiation exp	391.50	397.20	399.45	5.70	7.95

Full XPS scans of US 1–6 were performed, and the spectra are shown in the Supporting Information (Figures S1–S6). The full scan measurements show that only U, O, and C elements are on the surface.

**Figure 4:**
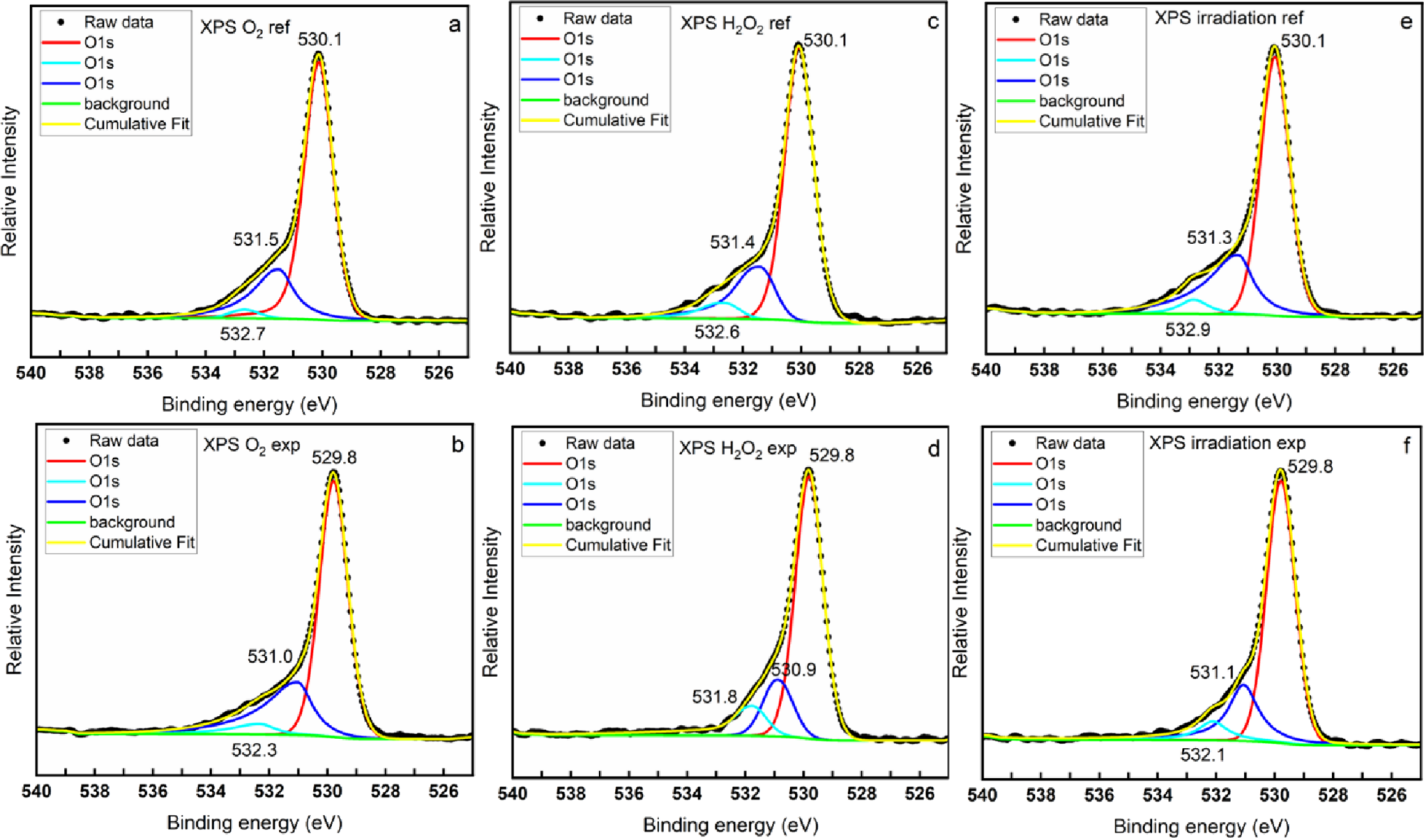
O 1s spectra of the UO_2_ slices exposed to various oxidizing conditions and reference samples with the deconvolution of the O 1s peak.

Uranium peroxide ((meta)-studtite) or hydroxide minerals ((meta)-schoepite) are common secondary phases formed on the UO_2_ surface.^38,40,41^ H_2_O_2_-induced oxidation through addition of H_2_O_2_ and water radiolysis generating H_2_O_2_ could potentially form (meta)-studtite or (meta)-schoepite with oxidized uranium. To elucidate the possible formation of these phases, narrow scans of O 1s spectra were performed on the UO_2_ surfaces after the exposures to oxidizing conditions. The results are shown in Figure 4. As can be seen, three peaks can be obtained from the deconvolution of the original O 1s peak. In the reference samples, the peaks are located at approximately, 530.1, 531.4, and 532.7 eV, with an FWHM of 1.2 ± 0.03 eV. The peaks at 530.1 eV are narrow in all cases and can be attributed to the oxygen in uranium oxides. The other two peaks have lower intensity and can be attributed to the hydroxyl group (531.4 eV) and carbonate group (532.7 eV). The carbonate group may come from the remaining experimental solution (10 mM HCO_3_^–^) that was not washed out during the rinsing process.^27^ Noteworthily, the CO_3_^2–^ peak is not observed in samples leached in solutions free from HCO_3_^–^ (not shown here). The O 1s peaks shift to lower binding energies (529.8, 531.0 ± 0.1, and 532.0 ± 0.3 eV) after exposure to oxidizing conditions with almost unchanged FWHM (1.2 ± 0.03 eV). The shift of the peaks indicates that the chemical environment surrounding the O atom is significantly changed upon oxidation from U(IV) to U(V). Interestingly, the narrow peaks, under all three exposure conditions, shift to 529.8 eV, representing a close stoichiometric ratio of O/U to each other. Comparing the shape of the narrow peaks at around 530 eV to the much broader O 1s peaks of uranyl peroxides reported in ref (42), formation of peroxide secondary phases can be ruled out. Generally, formation of peroxide or hydroxide secondary phases would lead to increased FWHM and changed peak positions due to the different chemical environment of the oxygen and the difference in crystal structure between the secondary phases and the oxidized UO_2_.^42^ So far, it is clear that the reduction in redox reactivity of a UO_2_ pellet (shown in Figure 1) is not due to formation of peroxide or hydroxide secondary phases but most probably due to the accumulation of U(V) on the UO_2_ surface.

**Figure 5:**
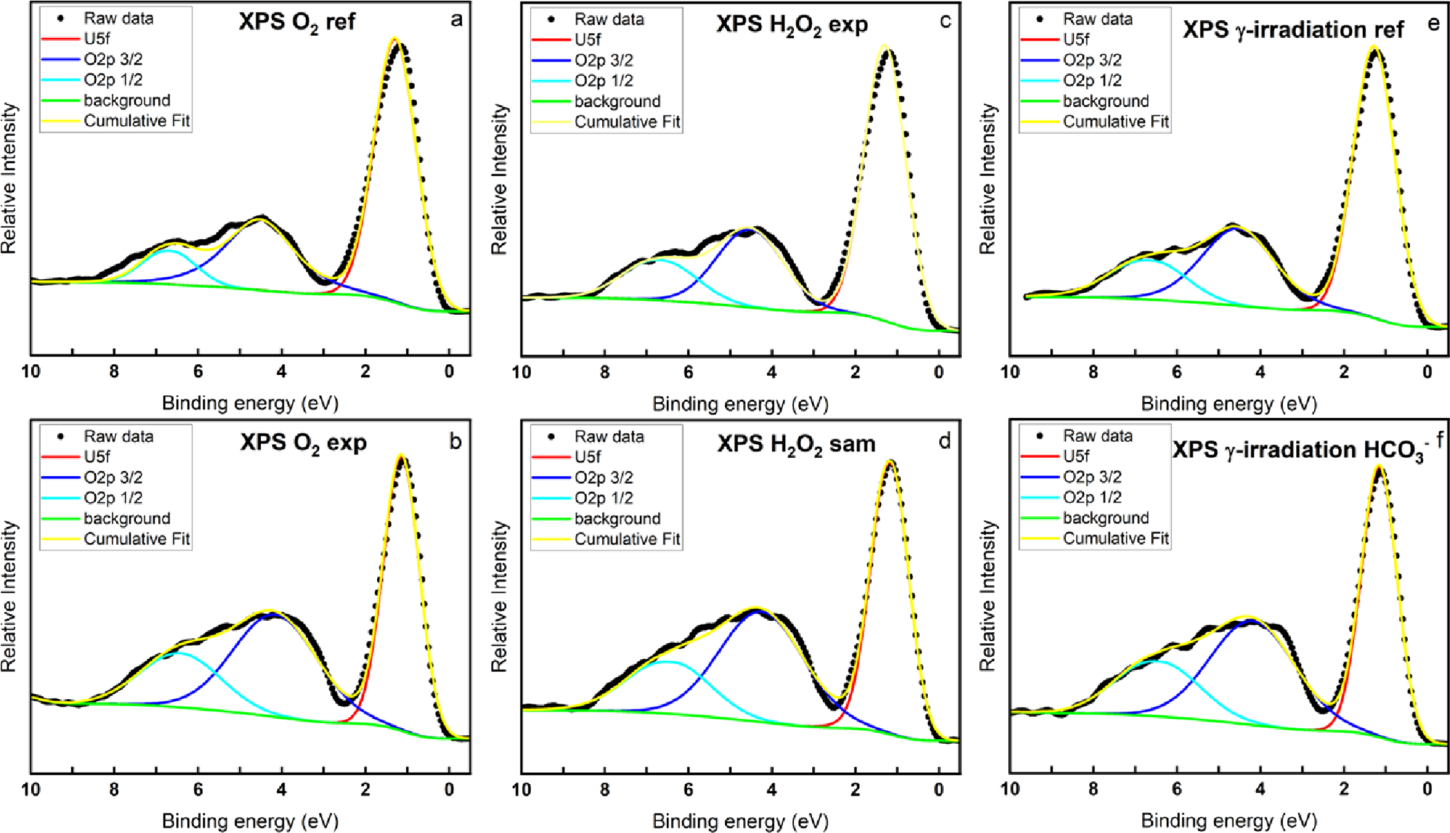
Valence band XPS spectra of the UO_2_ slices exposed to various oxidizing conditions and reference samples.

**Figure 6:**
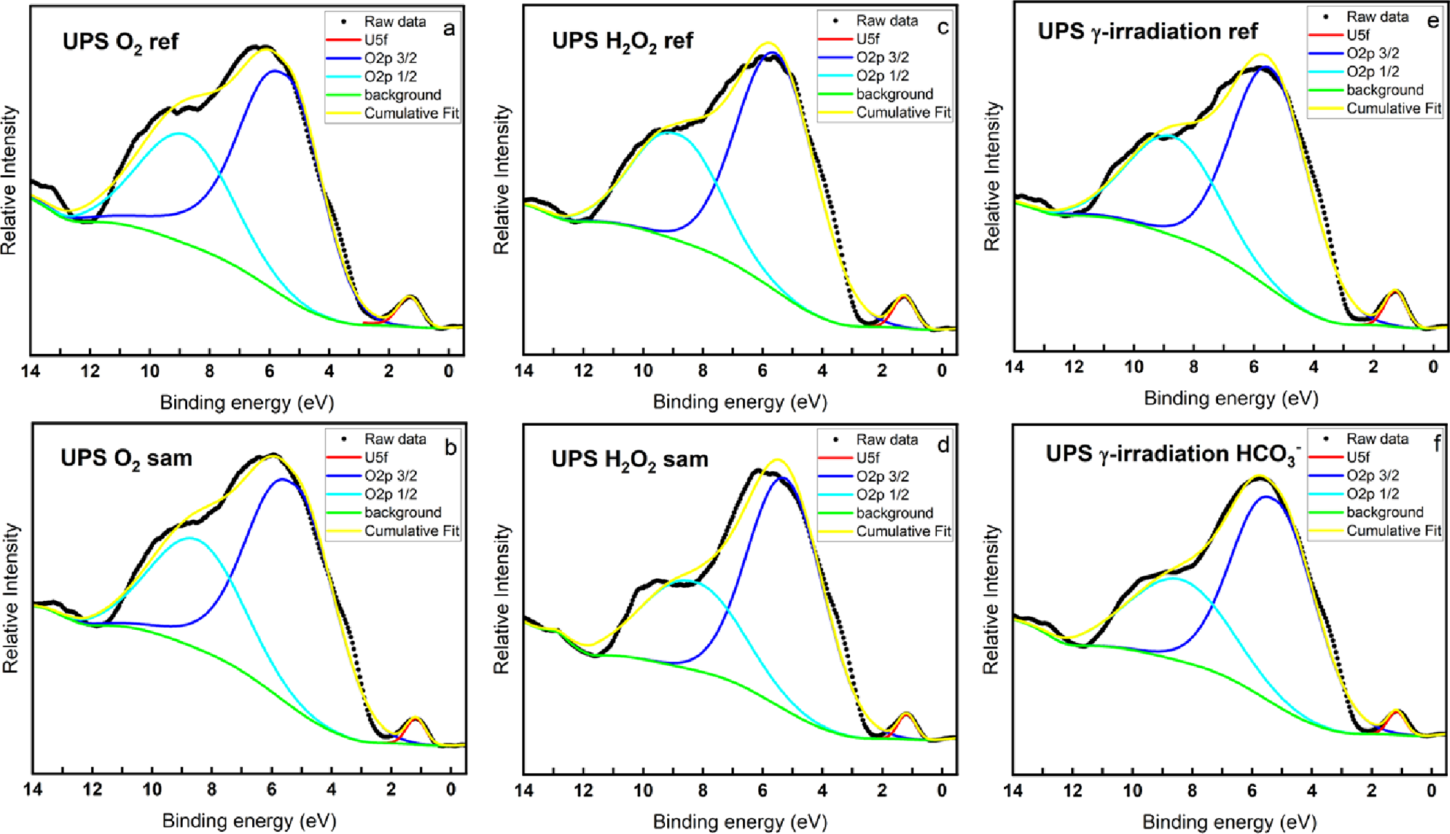
Valence band UPS spectra of the UO_2_ slices exposed to various oxidizing conditions and reference samples.

Figures 5 and 6 show the XPS and UPS measurements of the U 5f region (0–12 eV). Generally, XPS with an Al Kα source penetrates to a depth of approximately 10 nm (5 layers), whereas UPS with a He II source penetrates to a depth of approximately 1–2 nm (1 layer). In the figures, the sharp peaks at about 1.5 eV are the U 5f peaks. The electronic configuration of uranium in U(IV), U(V), and U(VI) are [Rn]5f^2^, [Rn]5f^1^, and [Rn]5f, respectively; therefore, when UO_2_ (U(IV)) is oxidized to U(V), the XPS and UPS spectra will display a decrease of the peak area of the U 5f peak. Also, no U 5f peak will be detected for pure U(VI) compounds. The broader peaks at 2–8 eV are the O 2p_1/2_ and O 2p_3/2_ peaks, the O 2p_3/2_ peak is at lower binding energy. The deconvolution is according to the principle that the peak area of O 2p_3/2_ should be twice as large as that of O 2p _1/2_ (corresponding to the spin-orbit splitting principle and 2 electrons in the 2p_1/2_ level, whereas 4 electrons in the 2p_3/2_ level).

It is worth mentioning that the O 2p_1/2_ and O 2p_3/2_ orbitals can hybridize with the U 6d and U 7s orbitals.^26,43,44^ Admittedly, accurate deconvolution and assignment of broad O 2p peaks into O 2p_1/2_ and O 2p_3/2_ and their hybridization (with U 6d and U 7s) peak are almost impossible due to the lack of relevant references. Since this work focuses on U(IV) to U(VI) oxidation states and there are no U 6d and U 7s electrons in uranium at oxidation states IV–VI, we only deconvoluted the broad O 2p peak to O 2p_1/2_ and O 2p_3/2_ peaks without taking the peaks for hybridization orbitals into account. In addition, the O 2p peaks for hydroxide and carbonate were not included in the peak deconvolution due to the lack of relevant references. The high FWHM values of O 2p_1/2_ and O 2p_3/2_ peaks and the poor match between the cumulative fit and raw data can most likely be attributed to the orbital hybridization effect mentioned above and the influence of the O 2p signal from OH^–^ and CO_3_^2–^ groups.

The area ratio of the U 5f peak to the O 2p_3/2_ peak is used to compare the oxidation states between the samples. The peak center, FWHM, and peak area of the deconvoluted peaks in the valence band UPS and XPS spectra as well as the area ratio between the U 5f peak and the O 2p_3/2_ peak are listed in Table 5. As can be seen, in both the XPS and UPS figures, the decreased area ratio of the samples exposed to oxidants compared to the corresponding reference samples indicates an increased oxidation state of the uranium. The decreased FWHM in the samples exposed to oxidants also indicates an increased oxidation state, and the narrowing of the U 5f peak is due to the change in the population of the U 5f orbital (U 5f^2^ to U 5f^1^ upon U(IV) oxidation to U(V)).^26^ Interestingly, the peak area ratio between U 5f and O 2p_3/2_ in all the UPS measurements is almost two orders of magnitude smaller than in the XPS measurements. Considering that the hyperstoichiometric UO_2.3_ obtained from the XPS U 4f deconvolutions is already close to pure U(V), only a combination of U(IV) and U(V) cannot reach such a small ratio between U 5f and O 2p_3/2_. Therefore, it is reasonable to assume that an ultrathin layer of the exposed pellets contains a significant amount of U(VI). Table 5 summarizes the FWHM and peak positions of the component U 5f, O 2p_3/2_, and O 2p_1/2_ peaks and the calculated peak area ratio between U 5f and O 2p_3/2_ for both XPS and UPS measurements.

**Table 5:** Summary of the U 5f Peak Position and the Deconvolution of O 2p as Well as the Area Ratio of U 5f and O 2p_3/2_

exposure condition	U 5f peak position	O 2p_3/2_ peak position	U 5f FWHM	O 2p_3/2_ FWHM	area ratio of U 5f to O 2p_3/2_
XPS O_2_ ref	1.29	4.52	1.18	2.02	3.73
XPS O_2_ exp	1.14	4.15	1.01	2.45	1.13
XPS H_2_O_2_ ref	1.30	4.51	1.18	1.93	2.20
XPS H_2_O_2_ exp	1.16	4.25	1.02	2.44	1.10
XPS irradiation ref	1.28	4.53	1.17	2.16	1.94
XPS irradiation HCO_3_^–^	1.16	4.18	1.01	2.46	1.08
UPS O_2_ ref	1.30	5.42	0.89	2.70	0.072
UPS O_2_ exp	1.18	5.20	0.75	3.00	0.025
UPS H_2_O_2_ ref	1.26	5.54	0.84	3.13	0.036
UPS H_2_O_2_ exp	1.17	5.23	0.68	2.96	0.025
UPS irradiation ref	1.27	5.44	0.82	3.05	0.043
UPS irradiation HCO_3_^–^	1.17	5.20	0.69	3.1	0.024

Based on the XPS results presented above, it is clear that the surfaces of the UO_2_ samples after exposure to oxidizing conditions in 10 mM HCO_3_^–^ are dominated by U(V) and without measurable amounts of U(VI). Numerous studies using electrochemical methods combined with surface characterization techniques such as XPS have demonstrated that, upon oxidation of UO_2_ in aqueous solution, the surface will first be oxidized to a U^IV^_1–2*x*_U^V^_2*x*_O_2+*x*_ layer followed by further oxidation to U(VI). Depending on the uranyl-complexing ability of the anions in solution, U(VI) will either deposit on the UO_2+*x*_ surface or dissolve.^45−52^ XPS results in these studies show that when HCO_3_^–^ is present, the oxidized surface was effectively U(V) with negligible amounts of U(VI). The latter being soluble is uranyl carbonate complexes. This is in line with our results i.e., formation of a U^IV^_1–2*x*_U^V^_2*x*_O_2+*x*_ layer with U(VI) dissolving in 10 mM HCO_3_^–^ leaching solution. In addition, Ulrich et al.^53^ investigated the stability of UO_2_ in 1 mM HCO_3_^–^ solution with dissolved oxygen (equilibrium with air). XPS results show that the surface layer contains 20% U(IV), 20% U(V), and 60% U(VI) after exposure. Interestingly, they also observed surface passivation of UO_2+*x*_ toward oxidative dissolution by O_2_ in carbonate solution. Again, the surface passivation as well as the presence of U(V) on the oxidized UO_2_ surface are in line with our observations. However, we did not observe the presence of U(VI) on the surface using XPS. This can most likely be attributed to the fact that, prior to XPS analysis, we rinsed the oxidized UO_2_ samples with pure water to remove soluble ions and complexes. In the work by Ulrich et al., the samples were dried in an airtight container without rinsing in pure water. Hansson et al.^29^ studied UO_2_ pellets exposed to radiation in aqueous solution under an Ar atmosphere for 45 days. XPS revealed that UO_2_ was oxidized to UO_2.33_ with no identified U(VI).

### Exposure to HCO_3_^–^ under Anoxic Conditions

Since UO_2_ will be oxidized to hyper-stoichiometric UO_2+*x*_ by atmospheric O_2_ during the dry storage prior to performing the experiments, the pellets and pellet slices were washed in HCO_3_^–^ solutions to remove oxidized uranium. In this work, there are three reference samples stored in 10 mM HCO_3_^–^ in a glovebox (O_2_ ≤ 0.1 ppm) for 45, 30, and 10 days, respectively. The storage time was determined by the exposure time to the oxidizing conditions of the corresponding experiment. The XPS measurements of the reference samples can provide some interesting information about the stability of oxidized UO_2_ in 10 mM HCO_3_^–^ solutions. As can be seen from Table 3, the calculated stoichiometric ratio of O/U of the reference samples is UO_2.11_ for the pellet slice exposed to 10 mM HCO_3_^–^ solution for 45 days, and UO_2.15_ and UO_2.17_ for the pellet slices exposed to the same solution for 30 and 10 days, respectively. The UPS data for these specimens indicate that the ultrathin layer of the reference samples is significantly oxidized. Since the oxidation of the ultrathin layer may very well occur during the rinsing of the pellet slice under ambient atmosphere or when transporting the pellet to the instrument for analysis, the actual oxidation state at the end of the exposure to 10 mM HCO_3_^–^ solution could be lower. The findings presented above imply that washing of a hyperstoichiometric uranium oxide surface to stoichiometric UO_2_ in anoxic 10 mM HCO_3_^–^ solution can take a substantial time. Hansson et al.^29^ also proposed that washing a hyperstoichiometric UO_2_ pellet in HCO_3_^–^ to a stoichiometry of UO_2.0_ is a slow process. This is quite interesting when revisiting some fairly recent work showing that a UO_2_ surface passivation phenomenon occurs after consecutive exposures to H_2_O_2_ in 10 mM HCO_3_^–^ solution.^23^ This passivation was suggested to be attributed to irreversible alteration of the pellet surface. However, it should be noted that the time to wash the pellet in 10 mM HCO_3_^–^ solution between exposures (24 h) was much shorter than the exposure times discussed above. It is therefore reasonable to suggest that the observed passivation to oxidative dissolution may not be irreversible. The recovery of the exposed surface upon exposure to 10 mM HCO_3_^–^ solution is only slow. The key question here is what the actual mechanism for dissolution of oxidized UO_2_ is. According to the XPS data presented above, the dominant form of oxidized uranium on the surface after exposure to oxidants is U(V). To the best of our knowledge, there are no reports on direct interactions between U(V) and HCO_3_^–^/CO_3_^2–^ or on direct dissolution of U(V). It has been shown that U(V) is a state within the fluorite lattice with charge compensation by interstitial O, while U(VI) usually forms a layered structure.^37,57,58^ Hence, the oxidation of U(V) to U(VI) involves a significant structural rearrangement, which probably has a direct impact on the kinetics of the process. U(V) is known to undergo disproportionation to produce U(IV) and U(VI) in solution.^54−56^ If this reaction is also possible on a solid surface (as has been proposed by Ulrich et al.^53^), this might explain why oxidized UO_2_ is slowly removed from the surface in the absence of an oxidant (i.e., U(VI) is removed once it is formed). The high proportion of U(V) on the surface and the slow reduction in U(V) content upon exposure to HCO_3_^–^ solution under anoxic conditions demonstrate the kinetic inertia of the process. It is interesting to note that in experiments where pure U(V) phases (U_2_O_5_) have been produced by reducing U(VI) (UO_3_), both the U(V) and the U(VI) phases have layered structures and reduction of U(V) to U(IV) involves a structural rearrangement. This structural rearrangement is also expressed in terms of kinetic inertia of the process.^30^ This deserves to be studied in more detail.

## Conclusions

The change in UO_2_ reactivity after consecutive exposures to either O_2_ or γ-radiation was studied. It was shown that the reactivity of UO_2_ decreased during O_2_ exposure in 10 mM HCO_3_^–^. The passivation phenomenon could not be observed for γ-radiation exposures since the system reaches a steady state. The surface of UO_2_ exposed to oxidizing conditions (O_2_, H_2_O_2_, and γ-radiation) in 10 mM HCO_3_^–^ was characterized by XPS and UPS. The XPS results show that the surfaces were significantly oxidized and dominated by U(V). Quantitative analysis was performed based on the deconvolution of the U 4f_7/2_ peak, and the stoichiometric ratio of O/U of the oxidized surfaces was calculated to UO_2.3_ for all the three oxidizing conditions. XPS measurements do not reveal any U(VI) on the exposed surfaces. However, UPS measurements indicate that the outer ultrathin layer contains a significant fraction of U(VI). Exposing the UO_2_ pellets to anoxic aqueous solutions containing 10 mM HCO_3_^–^ efficiently removes U(VI), while removal of U(V) is a much slower process. The actual reaction mechanism for U(V) removal by HCO_3_^–^ remains to be understood.

## Supporting Information Available

Details of the normalization of uranium dissolution experiments; full XPS scans of US 1–6; percents of different states of uranium in U 4f_7/2_ spectra deconvolution; discussion of U(VI) in U 4f spectra (PDF)
