# Supplementary material for: Exploring the Change in Redox Reactivity of UO2 Induced by Exposure to Oxidants in HCO3– Solution
Source: Inorg Chem. 2023 May 2;62(19):7413–23. doi: 10.1021/acs.inorgchem.3c00682 (PMC10189733; doi:10.1021/acs.inorgchem.3c00682)
Supplement: Supplementary file 1 — ic3c00682_si_001.pdf [file ic3c00682_si_001.pdf]

Supporting Information

Exploring the change in redox reactivity of UO_2 induced by exposure to oxidants in HCO_3^- solution

Junyi Li,*† Xianjie Liu‡ and Mats Jonsson†

†Department of Chemistry, School of Engineering Sciences in Chemistry, Biotechnology and Health, KTH Royal institute of Technology, SE-10044 Stockholm, Sweden.

‡Laboratory of Organic Electronics, Department of Science and Technology, Linköping University, Norrköping, SE-60174, Sweden.

Corresponding Author E-mail: ljunyi@kth.se

volume compensation (normalization)

The present work used the total dissolved amount of uranyl divided by the initial total $NaHCO_3$ volume (Equation 1). Where $[UO_2^{2+}]_n$ represents the normalized uranyl concentration after volume compensation, $[UO_2^{2+}]_m$ represents the directly measured uranyl concentration for each measurement. Vl is the solution volume left in the glass vessel after each measurement. The uranyl concentrations used in the rest of the text are all the normalized uranyl concentrations unless otherwise mention.

$$[UO_2^{2+}]_n = \frac{[UO_2^{2+}]_m \cdot V_l + \sum_{m-1}^{1}[UO_2^{2+}] \cdot V_{aliqout}}{V_0} \qquad (1)$$

Full XPS scans of US 1-6.

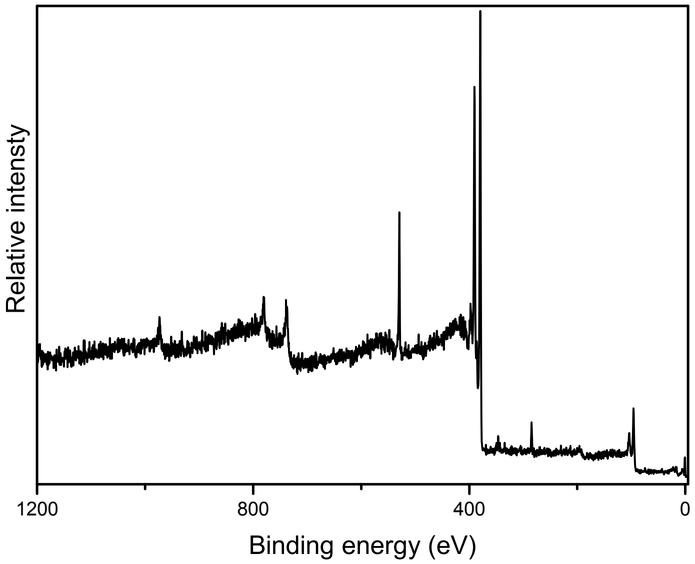

Figure S1. Full scan of UO_2 slice (US-1) after storage in anoxic 10 mM HCO_3^- for 45 days.

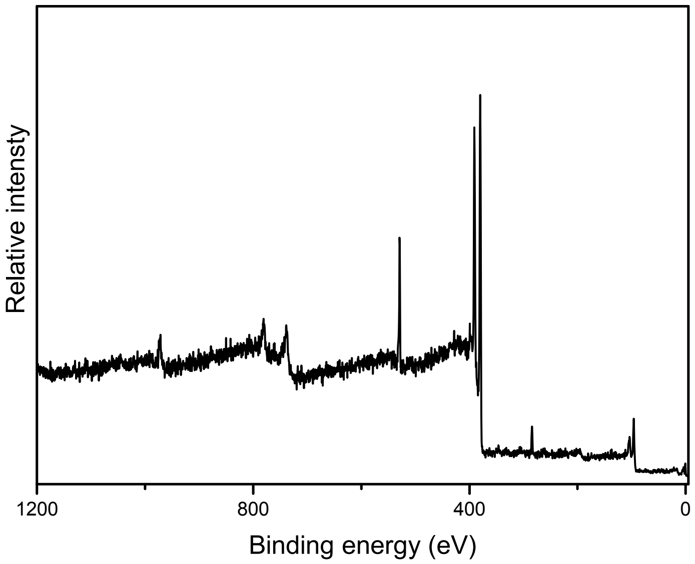

Figure S2. Full scan of UO_2 slice (US-2) after 3 consecutive O_2 exposures.

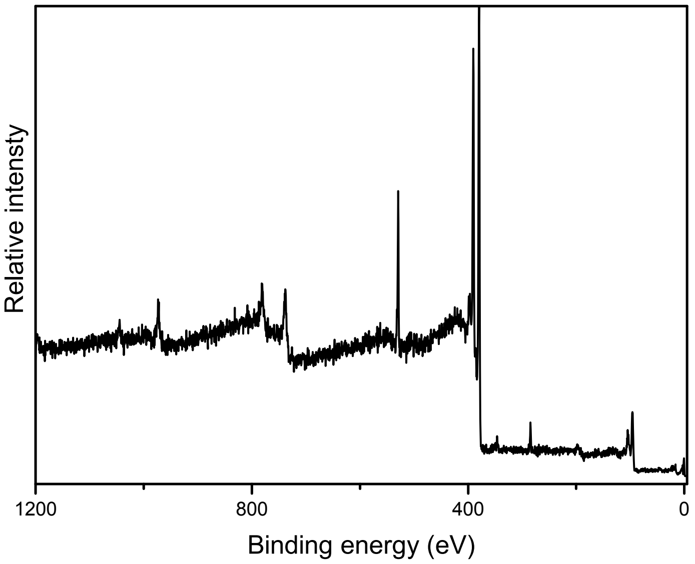

Figure S3. Full scan of UO_2 slice (US-3) after storage in anoxic 10 mM HCO_3^- for 30 days.

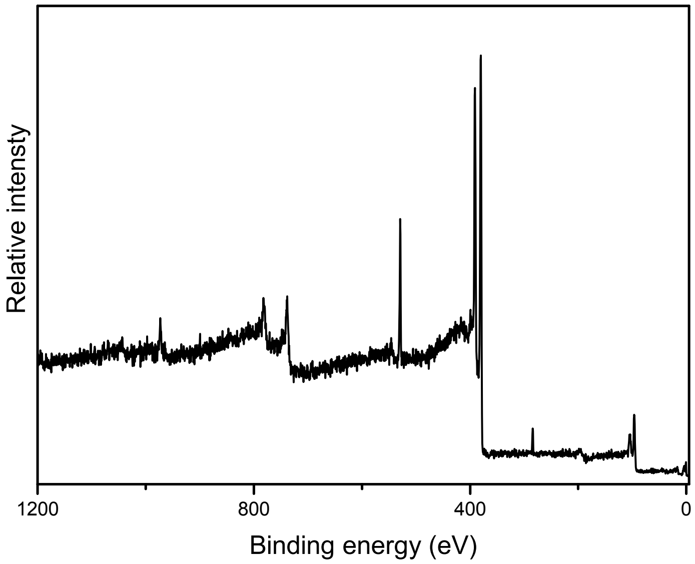

Figure S4. Full scan of UO_2 slice (US-4) after 3 consecutive H_2O_2 exposures.

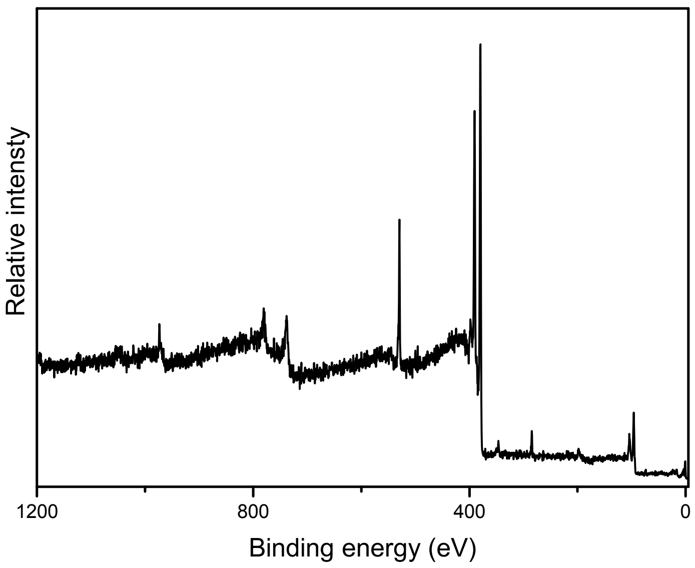

Figure S5. Full scan of UO_2 slice (US-$_5$) after storage in anoxic 10 mM HCO_3^- for 10 days.

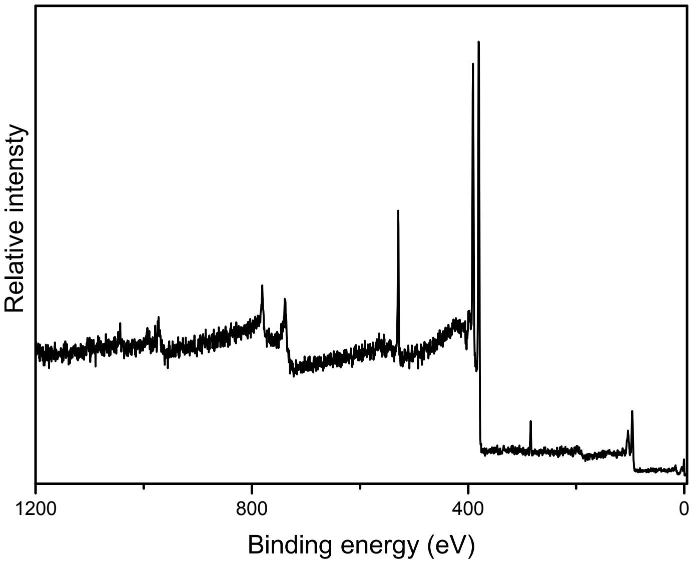

Figure S6. Full scan of UO_2 slice (US-6) after 3 consecutive γ - radiation exposures.

Table S1 Percents of different states of uranium in U $4f_{7/2}$ spectra deconvolution

Exposure condition	U(IV)	U(V)	U(IV)
O_2 ref	78%	22%	-
O_2 exp	38%	62%	-
H_2O_2 ref	71%	29%	-

H_2O_2 exp	35%	65%	-
Irradiation ref	67%	33%	-
Irradiation exp	37%	63%	-

Discussion of U(VI) in U 4f spectra

We forcibly added one peak and fixed the peak center at 382 eV with fixed FWHM (1.4 eV) and L/G ratio (80%). The parameters for U(IV) and U(V) used were the same as mentioned above (floating peak center but fixed FWHM, G/L and peak distance). The resulting spectrum is shown in Figure S7a. As can be seen, the component U(VI) peak area is almost negligible (3% of the peak area of U(V)), and the U(VI) peak is fully included in the U(V) peak. We then tried to fix the peak centers, FWHM, L/G of U(IV) and U(V) according to table 1, and allowed the U(VI) peak center float (with fixed FWHM and L/G). The U(VI) peak center will float to an unpredictable position in various spectra. An example is shown in Figure S7b. The peak position shifts to 381.6 eV and is still fully covered by the U(V) peak.

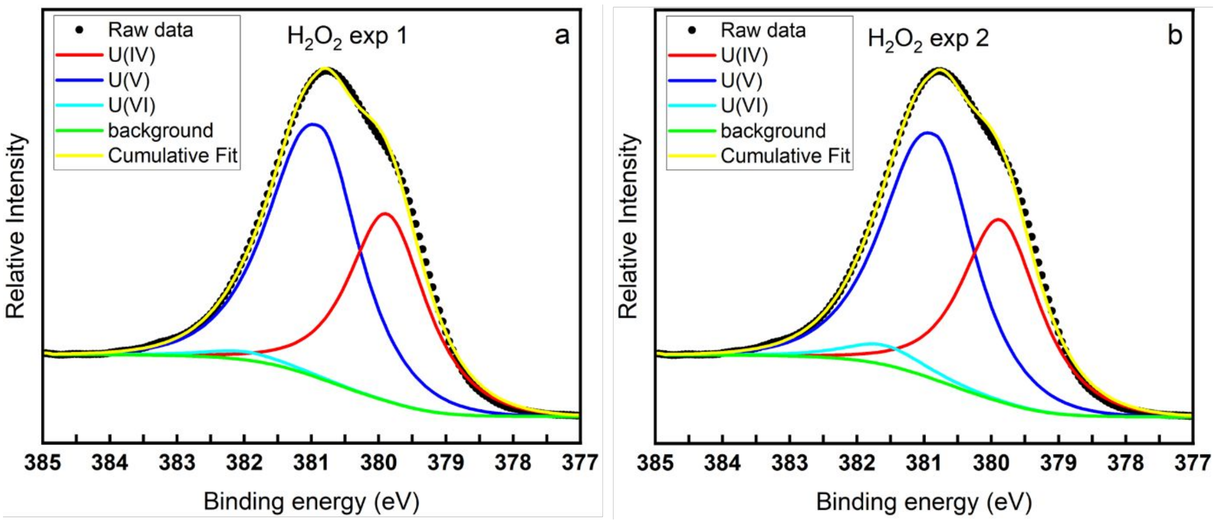

Figure S7. (a) Example of forcibly adding one U(VI) peak and fixed the peak center at 382 eV. (b) Allowing the forcibly added peak floating. The XPS raw data is from the sample after H_2O_2 exposure.
